# A comparison of post operative pain and hospital stay between Lichtenstein’s repair and Laparoscopic Transabdominal Preperitoneal (TAPP) repair of inguinal hernia: A randomized controlled trial

**DOI:** 10.12669/pjms.315.4811

**Published:** 2015

**Authors:** Umme Salma, Ishtiaq Ahmed, Sundas Ishtiaq

**Affiliations:** 1 Dr. Umme Salma, FCPS. Consultant Surgeon, Al-Nafees Medical College, Islamabad, Pakistan; 2 Prof. Dr. Ishtiaq Ahmed, FCPS. Consultant Surgeon, Al-Nafees Medical College, Islamabad, Pakistan; 3 Sundas Ishtiaq, Medical Officer, Al-Nafees Medical College, Islamabad, Pakistan

**Keywords:** Inguinal Hernia, Lichtenstein’s repair, Laparoscopic hernioplasty, Post operative pain, Hospital stay

## Abstract

**Objective:**

To compare the open Lichtenstein repair and laparoscopic mesh repair for direct inguinal hernias in terms of immediate post operative pain and length of hospital stay.

**Methods:**

This randomized control trial was conducted at Benazir Bhutto Hospital Rawalpindi from January 2009 to June 2010. All patients presenting in the surgical OPD with direct inguinal hernia, ASA I/II, were randomly divided in two equal groups. Group-I, patients underwent Lichtenstein’s repair and Group-II had hernioplasty by laparoscopic method (TAPP). Post operative pain intensity assessed by VAS and hospital stay measured in hours.

**Results:**

A total 60 patients of direct inguinal hernia were studied. The mean age was 61.48±7. The range of postoperative pain experienced was 5.55 as per VAS among all patients. In group-I (open hernioplasty) majority of patients (53.33%, n=16) experience severe type of pain where as in group-II, moderate severity of pain was reported by large number of patients (63.34%, n=19). The mean post operative pain intensity as per VAS was 6.23 in group-I and 4.43 in group-II patients. The mean length of hospital stay was slightly less (35.10 hrs) in group-I as compared to group-II (38.70 hrs).

**Conclusion:**

There is definitely less post operative pain after laparoscopic repair but hospital stay is same in both the procedures but laparoscopic procedure does increase the cost.

## INTRODUCTION

History of hernia repair is very rich and since ancient times surgeons have tried to improve it bit by bit. It is in fact a game of surgical anatomy, the one who understands the anatomy of Groin, can succeed in a way or the other to do a perfect repair. Herniorrhaphy is one of the commonest general surgical procedures performed and about 700,000 hernia operations are performed each year in the United States which is still on rise.^1^ Surgical outcome has improved tremendously due to improvements in surgical techniques, prosthetic materials and a better understanding of how to use them. Post operative pain, prolonged hospital stay and recurrence are a common problem associated with hernia surgery. Failure rate of less than 1% is reported from centers specialized in hernia surgery in contrast to much higher recurrence form non-specialized centers.^2^

Success of groin hernia repair is measured primarily by the permanence of the operation, fewest complications, minimal costs, and earliest return to normal activities. This success largely depends upon the surgeon’s competencies, preoperative patient selection and preparation, knowledge and experience of effective use of surgical techniques and currently available materials for repair.^1^ Endoscopic hernia surgery has increased significantly with the introduction of new operating techniques during the past decade. Day care open hernia surgery is routinely being performed in selected centers all over the world. Prolonged hospital stay and post operative pain are of more concern for patients immediately after surgery. Surgeons performing laparoscopic hernioplasty claim that there is decreased post operative pain and short postoperative hospital stay as compared to open hernioplasty.^3^,^4^ Anyway controversy persists regarding the most effective inguinal hernia repair. The aim of this study was to compare the open technique and the laparoscopic approach concerning post operative pain and hospital stay.

## METHODS

The objective of the study was to compare the open Lichtenstein repair and laparoscopic mesh repair for direct inguinal hernias in terms of immediate post operative pain and length of hospital stay. This Randomized controlled trial was conducted in Surgical Unit–I at Rawalpindi General Hospital, Rawalpindi for one year from January to December 2009. A total of 60 patients were studied and divided between the 2 equal groups. Patients were selected through Simple random sampling (computer generated) technique. Inclusion Criteria consist of male presenting to general-surgery clinic which are above thirty years of age with a diagnosis of direct inguinal hernia (acquired variety of inguinal hernia), Patients of ASA I & II category. Exclusion Criteria comprised of Patients with contraindications to pelvic laparoscopy, history of repair with mesh, recurrent inguinal hernia, previous pelvic surgery, history of Transvesical prostatectomy or patients having Hepatitis B or C positive. Routine baseline of all patients was checked. After obtaining approval by the hospital ethical committee, informed consent was taken from each patient. Pre-anesthetic evaluation was done before operation. All the procedures were performed by a single selected team of surgeons and assistants of the same surgical Unit. The patients were divided in two groups by simple random sampling (computer generated) to minimize the selection bias. No blinding possible.

### Group-I

Patients underwent hernioplasty by open method (Lichtenstein’s repair).

### Group-II

Patients underwent hernioplasty by laparoscopic method (TAPP).

Laparoscopic repair was performed by Transabdominal preperitoneal (TAPP) approach by 3 port technique, 1^st^ at the umbilicus, and the other two at lateral border of Rectus muscle at the level of umbilicus on both sides. Classical Open Lichtenstein’s repair was performed in the other group.

Postoperatively, patient’s perception of pain was assessed by Visual Analogue Scale (VAS) about four hours after surgery. All patients received analgesia in the form of Inj. Diclofenic sodium 75mg I/M immediately after surgery and it was repeated only after 06 hours. No preoperative or peroperative analgesia was given to any patient. All the patients were given standardized postoperative instructions not to restrict their activities unless the activities cause pain. All patients were assessed for postoperative analgesia requirements and hospital stay. Length of postoperative hospital stay was calculated in terms of hours after surgery till the time when patient was discharged. The discharge time was the time mentioned in patients’ notes.

**Fig.1:**
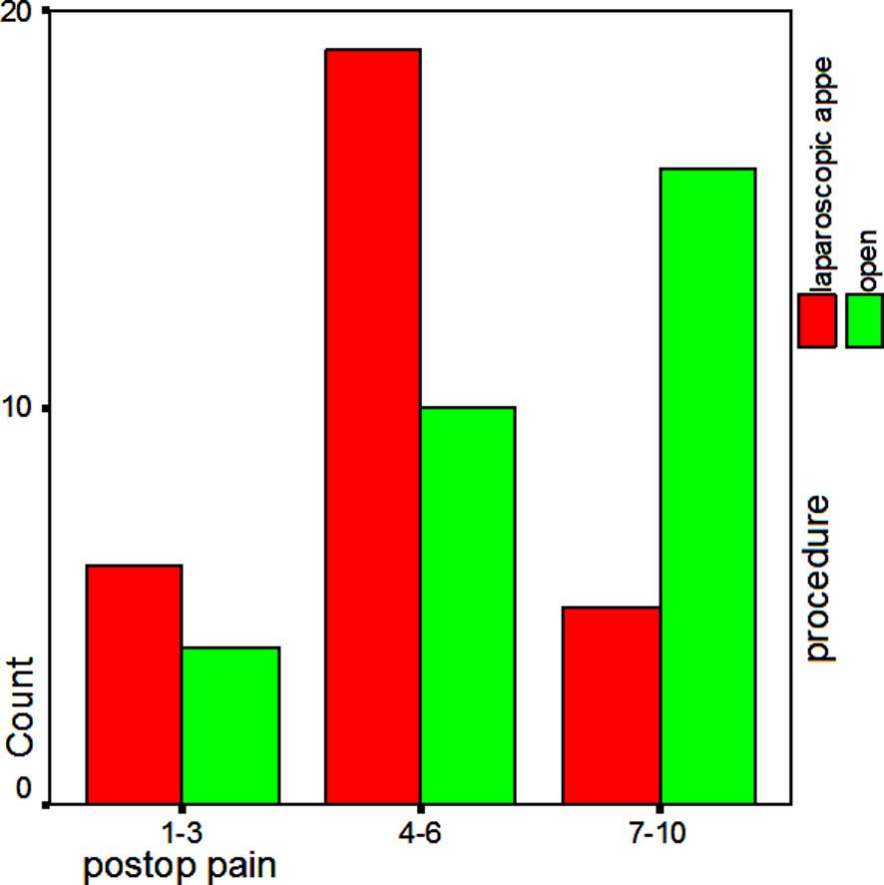
Comparison of severity of postoperative pain in laparoscopic and the open hernioplasty group.

All the data was entered on SPSS for windows version 10. Mean and standard deviation will be were calculated for all quantitative data (age, postoperative pain, length of hospital stay). Frequencies and percentages were calculated for qualitative data (age). Comparison of Quantitative data in both groups was analyzed by student T- test. Comparison of Quantitative data in both groups was analyzed by chi-square test in both groups. A p-value ≤ 0.05 was considered statistically significant.

## RESULTS

A total of 60 patients having direct inguinal hernia admitted through the surgical OPD from January to December 2009. The age varies between 35-75 years with a mean age of 61.48±7 years (Table-I). Patients were similar in demographic characteristics, all belonging to ASA I or II class. The range of postoperative pain experienced by the patients as per VAS was between 2-9 whereas mean was 5.55 (Table-II). The postoperative pain severity (ranked as mild, moderate and severe) showed severe type of pain experienced in 53.33% patients (n=16) in Group-I (open hernioplasty) followed by moderate severity in 33.34% (n=10) Patients. In group-II (Laparoscopic repair), majority of patients experienced moderate (63.34%, n=19) and mild (20%, n=6) severity of pain respectively. The mean post operative pain intensity as per VAS was 6.23 in group-I and 4.43 in group-II patients. The mean hospital stay was 36.90 hours and range was between 23 to 216 hours in both groups. (Table-III). Table–IV, shows that the mean length of hospital stay was slightly less (35.10 hrs) in group-I as compared to group-II (38.70 hrs).

**Table–I:** Severity of mean postoperative pain in all patients (n=60).

Postop pain	N	Minimum (VAS)	Maximum (VAS)	Mean	Std. Deviation
Valid N (listwise)	60	2	9	5.55	1.93

**Table-II:** Comparison of mean severity of Post operative pain in both groups (n=60), p-value 0.005.

Procedure Postop pain	N	Mean	Std. Deviation	Std. Error Mean
Group-I (Open)	30	6.23	1.87	0.34
Group-II (Laparoscopic)	30	4.43	1.59	0.29

**Table-III:** Mean Hospital Stay in all patients (n=60).

Hospital stay	N	Minimum	Maximum	Mean	Std. Deviation
Valid N (listwise)	60	23	216	36.90	25.71

**Table-IV:** Comparison of mean length of Hospital Stay in both groups (n=60) p-value = 0.592.

Hospital stay	Procedure	N	Mean	Std. Deviation	Std. Error Mean
	Open (Group-I)	30	35.10	12.55	2.29
	Laparoscopic (Group-II)	30	38.70	34.36	6.27

## DISCUSSION

The conventional surgery of groin hernias has been to ligate or reduce the hernia sac and reconstruct the posterior wall through an open incision. Although this operation can be performed as day care procedure in selected cases with the use of local anesthesia but it has been presumed that open hernioplasty is associated with increased postoperative pain, prolonged hospital stay, more recurrence and a delayed return (four to six weeks) to full physical activity and employment. The rates of hernia recurrence after open repair reported in literature are low (less than 2 percent) in specialized centers, but recurrence rates in regionalized studies of heterogeneous populations have averaged 5 to 10 percent for primary hernias and 5 to 30 percent for recurrent hernias.^2^ These problems with conventional herniorrhaphy along with the success of laparoscopic cholecystectomy provided the impetus to develop a laparoscopic approach to hernia repair.^1^ Laparoscopic inguinal hernia repair has been around since 1990s.^4^,^5^ Principal advantages of the laparoscopic approach over traditional surgeries reported in literature are, reduced postoperative pain, shorter hospital stays, and shorter periods of disability.^6^ The news media quickly portrayed laparoscopic surgery, with its small incisions, as a panacea (“minimally invasive,” “bandaid,” or “Nintendo” surgery), and the lay public demanded this form of surgery from its physicians and surgeons. Recently the single port robotic surgery for hernia is also used in specialized centers.^7^

In contrast with the open repair, Laparoscopic repair of inguinal hernias is performed with the use of general anesthesia and three laparoscopic ports. Several techniques for laparoscopic herniorrhaphy have been used, including closure or plugging of the hernia and various types of patch repairs. Patch repair is currently the most common method and entails placing a large prosthetic patch internally to cover the hernia and inguinal floor.^8^-^10^ Conceptually, this operation is similar to the open preperitoneal approach advocated by Stoppa et al., who used a large “tension-free” patch to cover the entire inguinal floor, with a subsequent recurrence rate of 1.4 percent.^10^ It appears, however, that laparoscopic hernia repair is associated with less postoperative pain and an earlier return to full physical activity than conventional herniorrhaphy.^11^,^12^ Despite the favourable early results, the procedure is controversial. Although the operation is similar to the repair described by Stoppa et al, the different method for fixation of the mesh laparoscopically adds an element of uncertainty to long-term stability and security.

Regarding post operative pain, it is reported in literature that the laparoscopic repair is associated with less pain as compared to open herniorrhaphy. The p value for postoperative pain is 0.005 in our study which is quite significant and concludes that the patient who had laparoscopic hernioplasty experienced less pain postoperatively as compared to those having open herniorrhaphy. The same results were also concluded from the review of 41 Cochrane studies,^13^ TULIP Trial^14^ and other studies.^15^ On the contrary, a multicenter trial conducted at Austria concludes no significant difference in complications and recurrence rate between laparoscopic and open hernioplasty.^16^ Similarly, a meta analysis conducted at Aberdine, UK conclude that the open and laparoscopic hernia repair are equally effective procedures and choice between them should be made on a case to case basis depending on patient preference and other characteristics such as age, work, health status etc.^17^ Many national and international studies also conclude no significant difference in morbidity and recurrence between both modalities but operating time is more in laparoscopic herniorrhaphy.^12^,^17^,^18^

Regarding hospital stay, our results shows that there is no significant statistical difference regarding postoperative hospital stay in either open or laparoscopic hernia repair. These findings are consistent with the many other studies carried out at different centers.^12^,^16^,^17^,^19^-^21^ and also with Cochrane database review of 41 studies.^13^ Literature search showed that there are many trials which have reported contrary results for example Pironi D et al.^22^, Neumayer et al.^8^ and Mahon et al.^6^ A recent audit published in 2009 have shown over all averaged 3.7 days hospital stay, averaging 3.3 and 3 days for bilateral and unilateral repairs respectively and any added procedures lengthened the hospital stay from 4 to 10.6 days.^20^

To date, recurrence rates with the laparoscopic preperitoneal prosthetic-patch operation have been low, but the follow-up has been short.^4^,^11^,^12^ Since most recurrences after conventional herniorrhaphy develop five or more years after the original operation, the long-term rates of recurrence may prove unacceptably high, especially when the procedure is performed by an inexperienced surgeon. A recent trial conducted by Brandt and his colleagues reported that the recurrence rate after 13 years of endoscopic total extra-peritoneal hernia repair is of 8.5% for primary and 10.8% in recurrent hernia with an overall 8.9% recurrence rate.^23^ Further evaluation in controlled clinical trials is therefore needed before laparoscopic herniorrhaphy is more widely implemented.

Laparoscopic hernia repair also requires a general anesthesia, with its associated risks, for a procedure that can be done conventionally with local anesthesia in selected cases. There is a small but definite risk of serious injury to intra-abdominal organs that is not associated with traditional inguinal herniorrhaphy. Also, costs may be higher because of the need for expensive equipment and other supplies related to laparoscopic instrumentation.^9^,^18^ Unlike those for laparoscopic cholecystectomy, these increased costs are not offset by decreased hospital charges, since hernia operations are routinely outpatient procedures regardless of the method of repair. A recent comparison of conventional with laparoscopic herniorrhaphy indicated an average increase in cost of 135 percent with the laparoscopic approach. Whether these direct costs may be partially offset by an earlier return to employment is not known.^18^,^22^

The safety and efficacy of laparoscopic inguinal hernia repair have recently been evaluated in two multi-institutional reports.^24^ In a multi-institutional trial, Fitzgibbons et al. reported 2.2 percent definite recurrences of hernia and 1.2% possible recurrences, whereas, in long term follow up, about 0.5% patients has reported thigh pain or hypoesthesia.^25^

## CONCLUSION

There is less post operative pain after laparoscopic repair but hospital stay is same in both the procedures. Keeping in view the limitations of Laparoscopic repair the choice between them should be made on a case to case basis depending on patient preference and other characteristics such as age, work, health status and cost etc.
